# A scoping review and quality assessment of machine learning techniques in identifying maternal risk factors during the peripartum phase for adverse child development

**DOI:** 10.1371/journal.pone.0321268

**Published:** 2025-05-28

**Authors:** Hsing-Fen Tu, Larissa Zierow, Mattias Lennartsson, Sascha Schweitzer

**Affiliations:** 1 Department of Women’s and Children’s Health, Uppsala University, Uppsala, Sweden; 2 Department of Psychology, Uppsala University, Uppsala, Sweden; 3 Department of Applied Educational Science, Umeå University, Umeå, Sweden; 4 ESB Business School, Reutlingen University, Reutlingen, Germany; 5 ifo Institute, CESifo, Munich, Germany; 6 Umeå University Library, Umeå, Sweden; 7 Faculty of Law and Economics, University of Bayreuth, Bayreuth, Germany; Children's Hospital of Los Angeles / Keck School of Medicine, UNITED STATES OF AMERICA

## Abstract

Maternal exposure to environmental risk factors (e.g., heavy metal exposure) or mental health problems during the peripartum phase has been shown to lead to negative and lasting impacts on child development and life in adulthood. Given the importance of identifying early markers within highly complex and heterogeneous perinatal factors, machine learning techniques emerge as a promising tool. The main goal of the current scoping review was to summarize the evidence on the application of machine learning techniques in predicting or identifying risk factors during peripartum for child development. A critical appraisal was also conducted to evaluate various aspects, including representativeness, data leakage, validation, performance metrics, and interpretability. A systematic search was conducted in PubMed, Web of Science, Scopus, and Google Scholar to identify studies published prior to the 14th of January 2025. Review selection and data extraction were performed by three independent reviewers. After removing duplicates, the searches yielded 10,336 studies, of which 60 studies were included in the final report. Among these 60 machine learning studies, a majority were pattern-focused, using machine learning primarily as a tool to more accurately describe associations between variables, while 16 studies were prediction-focused (26.7%), exploring the predictive performance of their models. For prediction-focused machine learning studies, a diverse range of methodologies was observed. The quality assessment showed that all studies had some important criteria that were not fully met, with deviations ranging from minor to major, limiting the interpretability and generalizability of the reported findings. Future research should aim at addressing these limitations to enhance the robustness and applicability of machine learning models in this field.

## Introduction

The peripartum phase, broadly defined as the period during pregnancy and up to 12 months after delivery [1], is a critical phase in children’s development [2,3]. Previous evidence shows that exposure to risk factors during this critical period (e.g., depression, alcohol, heavy metals) may result in structural and functional changes in the brain of neonates [4,5], and cognitive and neuropsychological development delays in infancy and early childhood [6,7]. The identification of early markers and predictors in the developmental pathway can help to improve personalized prevention programs and timely intervention. However, the influence of perinatal factors on child development is complex and often arises from the interplay of sociodemographic, psychosocial, biological, and environmental factors [7–10], making it challenging to identify clear risk factors and reliable predictors. Recent advancements in interdisciplinary research have enabled modern machine learning (ML) algorithms to effectively process large-scale, high-dimensional, and unstructured data [11]. In this review, we define ML as a set of algorithms that leverage heuristic, iterative optimization processes, thus adapting to data patterns without the constraints of requiring a predefined functional form or determining a closed-form solution. To capture the breadth of ML definitions in the literature, we distinguish between two categories of ML-based studies in this scoping review. Pattern-focused ML studies use machine learning methods to build models and gain insights into relationships among variables, but do not explicitly evaluate these models for predictive performance. In contrast, prediction-focused ML studies develop and assess models specifically for forecasting new data points. ML has been widely utilized by healthcare-related experts [12,13], epidemiologists [14], and developmental scientists [15] for predicting child outcomes. However, although research is increasingly focused on big data in recent years, challenges such as the lack of high-quality data and the transparency (often referred to as the “black box problem”) of ML approaches might reduce the quality of results and limit their interpretability [16]. Furthermore, critical appraisal of ML-related research, particularly in the form of review studies, is still uncommon. In this scoping review, we synthesized existing evidence from studies that employed ML approaches to examine maternal risk factors during the peripartum phase as predictors of child development. Moreover, we employed a novel assessment tool developed for evaluating the quality of ML research [17] to detect methodological challenges and limitations specifically in prediction studies.

### Potential maternal risk factors during the peripartum phase for developmental problems in offspring

It has been hypothesized that exposure to risk factors during pregnancy might shape the in-utero environment and change the developmental trajectories during the critical period of neurogenesis [18]. Additionally, the postpartum period, extending up to 12 months after delivery, is important as several maternal and family psychosocial risk factors (e.g., persistent depression, disharmony) [19,20], postnatal exposure to pollutants [21], and traumatic experiences [22], can elevate the risk for behavioral problems, depression, or academic challenges in children. Specifically, extensive research has shown that *internal risk factors* during the perinatal phase, such as perinatal mental health problems and adverse traumatic childhood experiences, are related to offspring’s internalizing and externalizing problems [23], social-emotional, cognitive, and motor development [6,7,24,25]. Moreover, studies have reported that perinatal mental distress and the use of selective serotonin reuptake inhibitors (a type of antidepressant drug) might cause disturbances in fetal brain development and increase the risk of neurodevelopmental disorders [4,26–28]. For *external risk factors*, it has been shown that toxin exposure [29,30], even prior to pregnancy, may alter a child’s developmental trajectories, and may affect brain structure in early childhood [31]. Research focused on environmental factors has demonstrated the long-term adverse effects of prenatal exposure to various metals on offspring’s cognition, intelligence quotient (IQ) [32], self-regulatory function, learning difficulties [33], and more. Moreover, emerging evidence indicates that prenatal exposure to alcohol [34], chemical substances [35], air pollution [32], nutritional problems [36], and smoke [37] might elevate the risk of neurodevelopmental disorders (e.g., attention deficit/hyperactivity disorder and autism spectrum disorder) in children. Though the etiologies of attention deficit/hyperactivity disorder (ADHD) and autism spectrum disorder (ASD) are still unclear, several epigenetic studies have provided evidence that perinatal exposure to these internal and external risk factors is associated with an increased risk for ADHD or ASD [38,39]. In recent years, the increasing prevalence of ADHD [40] and ASD [41] has brought more attention to early detection and intervention. Furthermore, besides risk factors during the peripartum period, research focusing on cross-generational transmission has reported that fetal brain and child outcomes may be vulnerable to a mother’s adverse experiences (e.g., maternal childhood maltreatment, experience of stressful legal problems) or substance exposure prior to pregnancy [42–45]. Together, these findings underscore the complex interplay between various risk factors, making it a major challenge to achieve early identification of developmental problems and identify early predictors. This complexity also hinders efforts to support diagnosis, prevention, and timely intervention.

### Applications of machine learning techniques in identifying maternal risk factors during the peripartum phase for adverse child development

ML provides insights into large amounts of data using robust probabilistic techniques. One of the main advantages of ML is its ability to incorporate interactive predictors and account for variables with non-linear relationships. To address the challenges posed by complex and heterogeneous data in predictive modeling, ML techniques have emerged as a promising tool for supporting the diagnosis of psychiatric disorders [46,47], cancers [48], and ASD [49]. Furthermore, extensive research has shown that ML can utilize perinatal data to assist in predicting fetal health, neonatal outcomes (e.g., birth weight), preterm birth, stillbirth, length of intensive care, and mortality [17,50–54]. In the context of perinatal risk factors and clinical outcomes of the child, several previous studies have focused on prenatal exposure to alcohol [55,56], cytokines [57], toxins and metals [58], or chemical compositions [59], and have investigated the various prediction models of developmental disorders, such as ASD or ADHD. While the application of ML for prediction is considered as a promising tool in many fields, potential pitfalls related to biases in the data, data quality, and the lack of standardized evaluation of prediction performance, as well as generalizability and its transfer into practice, have been discussed [15,16]. With the increasing adoption of ML applications in the field, several systematic reviews have examined their use in prediction models for pregnancy outcomes [60], preterm birth [61], neurodevelopmental outcomes after preterm birth [17], and children’s cognitive outcomes [15]. However, there is a notable gap in synthesizing the evidence on the application of ML to predict later child development based on maternal risk factors in non-preterm or extremely low birth weight infants. This gap exists partly because full-term infants are often considered at lower risk compared to preterm or extremely low-birth-weight infants, who are more closely monitored and frequently included in predictive studies. Furthermore, although guidelines for ML applications exist [62], an encompassing evaluation of the quality of evidence required to overcome methodological challenges is often lacking [17].

### This scoping review

This scoping review summarizes studies focusing on the application of ML techniques for describing or predicting child development based on maternal risk factors during the peripartum phase, covering full-term and healthy-born children. While preterm and low-birth-weight infants often receive early interventions due to known risks, identifying risk factors or predictors of developmental delay in full-term healthy infants is challenging. Additionally, a critical appraisal was performed, evaluating several aspects, including representativeness, data leakage, internal and external validation, performance metrics, and interpretability. This review provides an overview of the current research on predicting child development outcomes, highlights gaps in the literature, and suggests directions for future studies to enhance the applicability of ML techniques in this context.

## Methods

The current review was not pre-registered publicly in a repository. For an overview of search results and information extracted from the studies, please see the following link on the Open Science Framework (https://osf.io/n5gyd/).

### Search strategy

Systematic searches were conducted using PubMed, Web of Science, Scopus, and Google Scholar to identify published studies. The literature search process was carried out with the assistance of two senior librarians, one of whom is a co-author. The searches were restricted to articles published up to January 14, 2025. Keywords related to maternal factors included perinatal, antenatal, prenatal, postnatal factors, postpartum, and pregnancy. Keywords related to child development included infant outcome, motor development, cognitive development, regulation, social-emotional development, and neurodevelopment. Other keywords included terms such as ML, artificial intelligence, artificial neural networks, Bayesian kernel machine regression (BKMR), decision tree, deep learning, k-means, neural network, random forest, regression tree, supervised learning, semi-supervised learning, and unsupervised learning. These keyword groups were used in a Boolean search across all four databases. Additionally, a MeSH search was conducted in PubMed and a topic search was performed in Web of Science and Scopus to ensure broader coverage of potentially related content (see S1 Table). After the search, we used Rayyan [63] to support the removal of duplicates before initiating the screening process.

### Inclusion criteria for study eligibility

This review follows the PRISMA guidelines (Preferred Reporting Items for Systematic Reviews and Meta-Analyses, S2 Table). The relevance of the identified articles was evaluated through a three-step process. In the first step, authors assessed the articles’ titles, then reviewed the abstracts in the second step, and finally, examined the full articles in the third step. Each step was independently completed by two authors. To maximize the number of articles included, when evaluating the articles, the inclusion criteria were applied conservatively, meaning that the articles were retained as long as one author chose them. All articles at each step were evaluated based on the following inclusion criteria: (1) journal article in English, (2) original study (not review or commentary), (3) use of artificial intelligence or ML in their methodology (as defined in the Introduction), (4) use of maternal risk factors during the peripartum phase (e.g., psychosocial problems, or environmental exposure) as independent variables, and (5) use of children’s developmental outcomes (e.g., cognitive, social-emotional, motor development, or the presence of neurodevelopmental disorders) as dependent variables. Case studies and book chapters without empirical study results were excluded. Studies focused on fetal development, birth outcome (e.g., preterm, very low weight, still birth, mortality), or physiological health related outcomes (e.g., allergy, thyroid function, and congenital heart disease) were also excluded. Additionally, children born through in vitro fertilization, which is not in the scope of the current review, were excluded. Neural imaging studies, such as those using electroencephalogram or magnetic resonance imaging, were also excluded due to their complexity and challenges in data interpretation compared to more accessible and interpretable methods, such as questionnaire-based data and electronic health records. An overview of the inclusion and exclusion criteria is provided in Table 1.

**Table 1:** Overview of Inclusion and Exclusion Criteria.

Inclusion	Exclusion
Journal article in English Original study Human subject Use of ML in methodology Use of maternal exposure to risk factors during peripartum phase as independent variables Use of infants’ outcomes (cognitive, social-emotional, motor development, regulation, and neurodevelopmental outcome, such as ADHD or ASD)	Review, protocol, or case report Outcome measures during the fetal phase (before birth, such as fetal size) Preterm and very low-weight infants Infants born with congenital issues, genetic neurological disorders (e.g., Down Syndrome), or brain malformations Stillbirth or mortality rate as outcomes Physiological health-related outcomes (e.g., allergy, thyroid function, congenital heart disease) Assisted pregnancy (IVF or ICSI) Imaging outcome at the neural level or cellular level, such as fMRI or DNA data Outcome measured in the late adolescent phase (or young adulthood)

Abbreviations: ADHD: Attention-Deficit/Hyperactivity Disorder; ASD: Autism Spectrum Disorder; DNA: Deoxyribonucleic Acid; fMRI: Functional Magnetic Resonance Imaging; ICSI: Intracytoplasmic Sperm Injection; IVF: In Vitro Fertilization; ML: Machine Learning.

### Screening and data extraction

We performed three steps to screen titles, abstracts, and full texts based on the inclusion and exclusion criteria. After screening full texts, information such as publishing details, study population, data source, perinatal risk factors, timing of exposure to risk factors, child outcome assessments and timing, ML techniques, other statistical approaches for validation, and main findings was systematically extracted. All procedures were executed by at least two authors, and conflicting assessments were resolved through consensus-based discussions.

### Quality assessment

The current scoping review used the framework developed by Boven et al. [64]. Zierow, Tu, and Schweitzer extracted information related to six different criteria, including *participants (sample size), data leakage, validation, performance metrics, interpretability,* and *open science*. Note that these criteria depend on each other sequentially. First, only a sufficient number of observations allows for an appropriate split of the dataset for validation purposes. Second, a clean splitting strategy and caution in the tuning of model parameters ensures that the validation is meaningful. Third, only if the previous criteria are fulfilled do performance metrics become meaningful. In a similar vein, transparency and proper documentation are prerequisites for fully assessing the studies. Specifically, this is captured by the criterion of interpretability and open science in the quality assessment.

For sample size and participants, several hundred or more participants are considered appropriate. For data leakage, it is evaluated whether inflated performance due to overfitting has been introduced. For validation, the use of a completely independent sample for external validation is considered indicative of high quality. For the performance metrics, it is evaluated whether metrics such as the area under the receiver operating characteristics curve (AUC) and base rate-sensitive metrics are reported. For interpretability, it is evaluated whether authors provide additional insights into the models, for example, by conducting a comparison with previously developed models. Finally, for open science, high quality is indicated by a study’s support of transparency of data, codes, and models, which would allow for the future reproduction of the methodology. Each parameter was rated as appropriate (score = 2), minor deviation (score = 1), or major deviation (score = 0). The total score ranges from 0 to 12 with a higher total score indicating a higher quality study. In addition to calculating the total score for each study, the scores were also aggregated for each of the six criteria across all included predictive studies, providing a more detailed overview of performance in specific areas.

## Results

**Fig 1:**
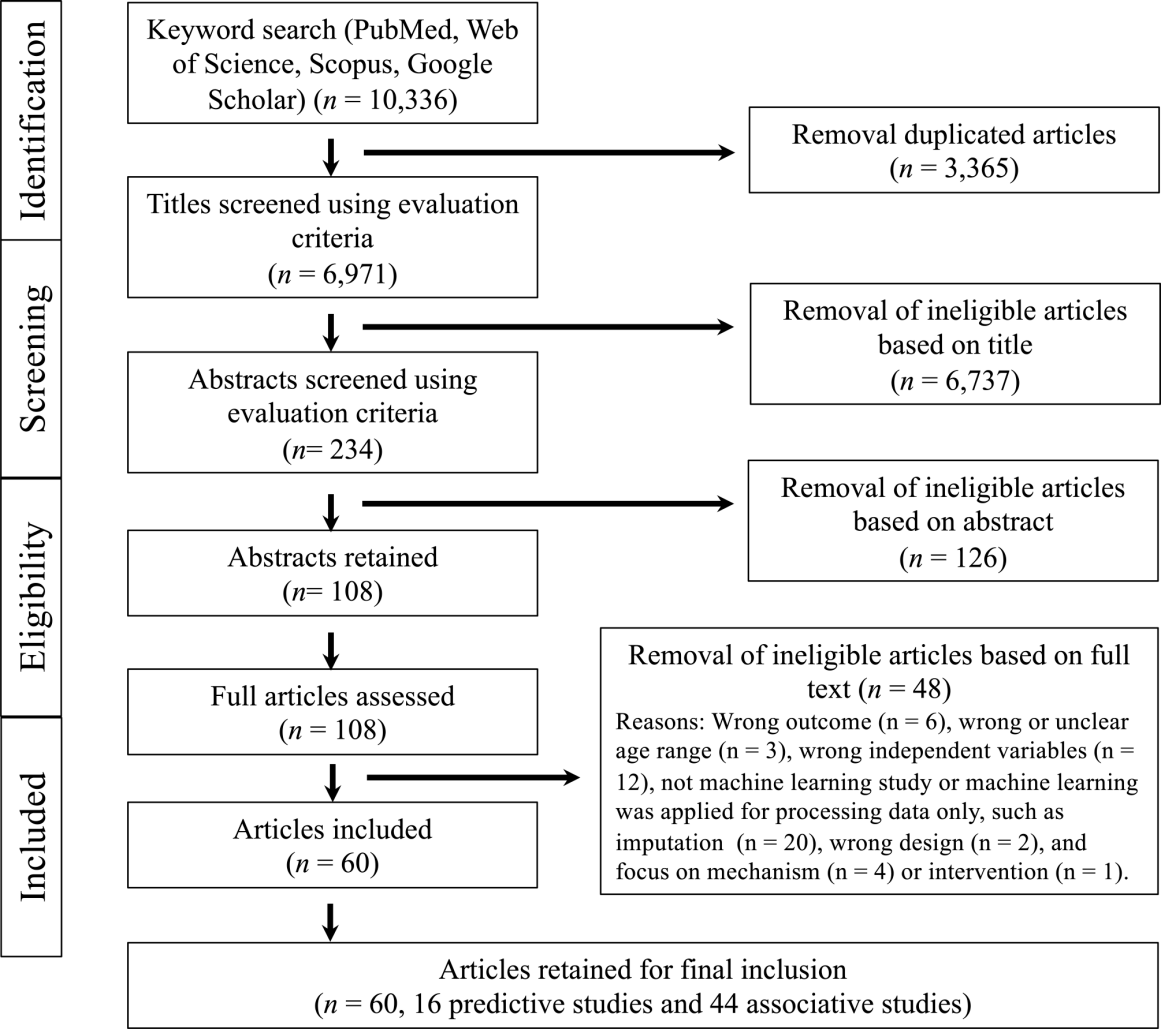
PRISMA flowchart illustrating the study selection process for the scoping review. A total of 10,336 articles were identified through database searches. Duplicate records were removed using Rayyan, resulting in a unique set of articles. The screening was conducted in three steps (title, abstract, and full-text review), each independently performed by 2–3 authors. Following this rigorous selection process, 60 studies were included in the final review.

The search resulted in 10,336 identified studies, of which 3,365 were duplicates. After screening and removing ineligible studies, 60 studies were retained in the current review. The PRISMA flow diagram [65] depicting the selection of studies is presented in Fig 1. Tables 2 and 3 summarize the study characteristics and main findings. For those studies with prediction models (prediction-focused ML studies), results of the quality assessment are reported in Table 2. Among the 16 studies included in this subgroup, the populations were from Ukraine, Australia, Sweden, France, Italy, Iran, Turkey, Brazil, Korea, and China (each with one study), while Israel, Ireland, and the USA each had two studies. These studies examined various outcomes, with neurodevelopmental diagnoses accounting for the largest proportion (40%), while cognitive and motor development each comprised 30% (S1 Fig). The detailed rating for the quality assessment of each of the quality criteria in each study is reported in the S3 Table.

**Table 2:** Characteristics of prediction-focused ML studies.

First author	Year	Country	Data source	Sample size	Maternal exposure to risk factors/ predictors	Timing of exposure	Child outcome assessment	Age of assessment^1^	Main machine learning technique	Other^2^ statistical approach for robustness	Main findings	Quality level
**Balaraman**	2016	Ukraine	Cohort subset, CIFASD.org Longitudinal study Western Ukraine	68	Alcohol exposure as main risk factor; miRNAs as main predictors; other predictors, such as smoke and SES	Prenatal (2^nd^ & 3^rd^ trimesters)	BSID-II, MDI, PDI, FASD	Birth, 6m, and/or 12m	RFM	ANOVA	In a random forest analysis classification model, a combination of high variance miRNAs, smoking history and socioeconomic status classified membership in the “exposed and affect group” and the “unexposed” group, with a misclassification rate of 13%. The RFM classified 17% of the “exposed but unaffected” group as unaffected, whereas 83% were “exposed and affected”, at least at one stage of pregnancy.	5/12
**Ben-Sasson**	2024 [66]	Israel	MoH Well-baby Clinics cohort	608,079	Postpartum depression was included in postnatal featured predictors	Postnatal	Likelihood for ASD (ca. 2.1%)	6 weeks-6 y	Gradient boosting decision tree	None	Among all predictors, postpartum depression was not included in feature importance plot in both boys and girls. Instead, “parent concern for development” showed the highest values in both boys and girls. Note: preterms were included in this study.	8/12
**Ben-Sasson**	2024 [67]	Israel	MoH Well-baby Clinics cohort	780,610	Postpartum depression was included in postnatal featured predictors	Postnatal	ASD diagnosis	≥ 2y	Gradient boosting decision tree, LR	Naïve Bayes	Postpartum depression was not included in feature importance plot. There were several other perinatal-related factors, such as parent concern for development, mother age, pregnancy week were included.	9/12
**Betts**	2023	Australia	The New South Wales data collection; the New South Wales admitted patient data collection, the New South Wales mental health ambulatory data collection	262,650	Various of perinatal factors, including diseases in pregnancy, mental and nervous system disorders in pregnancy, tobacco use, depressive episode, intrapartum hemorrhage, etc.	Prenatal period, delivery, and up to 12 months postpartum	ASD diagnosis based on ICD 10	14 y	LR with elastic net regularization and gradient boosting trees	None	The most effective model achieved an area under the receiver operating characteristic (ROC) curve of 0.73 in predicting ASD. Key risk factors identified for the diagnosis included offspring gender, maternal age at birth, use of delivery analgesia, maternal prenatal tobacco use disorders, and a low 5-minute APGAR score.	8/12
**Bowe**	2022	Ireland	Irish population-based BASELINE cohort	1,080	Smoking during pregnancy, maternal depression, alcohol consumption in the 1^st^ trimester	Pre-, peri, and postnatal	KBIT-2	5 y	RFM, LR	None	The full random forest model using the complete set of 21 predictors achieved an accuracy of 95%, sensitivity of 89%, and specificity of 99% in predicting IQ scores ≤ 90. The most important predictors include total years of maternal schooling, infant Apgar score at 1 min, socioeconomic index, maternal BMI, and alcohol consumption in the first trimester.	7/12
**Bowe**	2024	Ireland	Growing Up in Ireland cohort	8,858	Alcohol intake	Postnatal	Cognitive ability based on BAS	5 y	RFM, SVM, LR^3^	None	The authors reported that prenatal exposure to the air pollution mixture was linked to lower general memory and attention/concentration scores, suggesting poorer memory function. Additionally, it was associated with more omission errors on the CPT-II test, indicating increased attention problems.	8/12
**Brynge**	2022	Sweden	National register-based (Stockholm Youth Cohort)	747	Exposure to cytokines	Prenatal (1^st^ trimester)	Diagnose of ASD	≥ 4y	RFM	PCA & pairwise univariate regression	Authors find no evidence to suggest that adding these cytokines and other markers of maternal immunity, to register-based maternal factors (e.g., psychiatric history) improves prediction of ASD.	7/12
**Caly**	2021	France	Maternity Hospital of the University of Limoges	252	Potential risk factors from maternal and embryo’s bio- and physiological data	Prenatal (3^rd^ trimester) and birth	Diagnose of ASD	4-5y	Gradient boosting decision tree	Linear regression; ANCOVA	Authors report that a combination of the collected features during maternity and during birth impacts the classification and prognosis of ASD, including some features that are not intuitively linked to ASD.	4/12
**Goh**	2016	USA	Collaborative Initiative on Fetal Alcohol Spectrum Disorders (CIFASD)	888	Alcohol exposure	Prenatal	Diagnose of ADHD, CBCL, WISC-IV/DAS-II, VABS	8-16y (n = 434); 5-7y & 10-16y (n = 454)	DT, LR	None	The decision tree model consisted of variables from 2 parent questionnaires, an IQ score, and a physical examination showed accuracy rates for both the development and validation samples with 80% overall accuracy.	8/12
**Grossi**	2016	Italy	Clinical sample	137	Exposure to toxics: paints, lacquers, paint thinner solvents, detergents, disinfectants, toluene, benzene, phenol, and metal (in particular chromium), “Polyvinyl chloride flooring”	Prenatal	Diagnose of ASD based on the ACARS	12-13y	Supervised ANN, LR	None	Specialized ANNs discriminated between autism and control subjects with 80.19% global accuracy when the dataset was preprocessed with training with input selection and testing system selecting 16 out of 27 variables. Logistic regression applied to 27 variables gave unsatisfactory results with global accuracy of 46%.	5/12
**Li**	2023	USA	Subset of a cohort with women who reside in the USA and Canada	82	Chemical composition milk sample (Mothers with depression, diabetes, or pregnancy complications were excluded)	Prenatal	ASQ2	14.2 ± 3.1m (range 12-25m)	RFM	None	Authors report sex-specific, multi-analyte predictive models from the human milk metabolome and exposome to predict future risk of neurodevelopmental delay in the toddler age with over 75% accuracy.	4/12
**Soleimani**	2013	Iran	Medical record	1,232	Pre-, peri-, and post-natal biological hazards that can compromise development	Prenatal, birth, and postnatal	INFANIB	35.77 ± 15.77w	ANN	Linear regression	Authors report that true prediction of developmental disorder in the artificial neural network model, compared to the logistic regression model, was 83.1% vs. 79.5% and the area under ROC curves, calculated from testing data, were 0.79 and 0.68, respectively. In addition, specificity and sensitivity of the ANN model vs. logistic model was calculated 93.2% vs. 92.7% and 39.1% vs. 21.7%. An ANN performed significantly better than a logistic regression model.	8/12
**Usta**	2020	Turkey	Early Childhood Mental Health Profile	2,775 (only 106 included in the ML models)	Maternal psychopathological symptoms measured by the Brief Symptoms Inventory and the Psychiatric Evaluation Form	Postnatal	ASQ, BITSEA	12 – 42m	DT, SVM, LR	Linear models, Naive Bayes	A total of 106 children were identified as at risk, as they were above the clinical cut-off point (1.5 standard deviations) of the BITSEA points and below the cut-off points of any one of the developmental areas of the ASQ. Modeling was applied to the data of these 106 children. The Support Vector Machines (SVM) model was selected for prediction with the automatically optimized highest AUC value (0,75), accuracy 90.8%, sensitivity 94.0%., and specific 74.3%.	6/12
**Viegas da Silva**	2024	Brazil	Pelotas Birth Cohort	3,603	27 potential predictors, e.g., prenatal and postnatal depressive symptoms, adverse childhood experiences, diabetes, drug, and alcohol use during pregnancy	Prenatal and postnatal	BDI	4y	Combination of DT and linear regression	PCA	Maternal schooling was the strongest predictor of BDI, followed by paternal schooling, with lower parental education linked to lower BDI. Higher family income was associated with better scores. Depressive symptoms in mothers and maternal adverse childhood experiences combined with alcohol use during pregnancy were linked to lower BDI. These findings highlight the importance of parental education and early-life experiences in child development.	10/12
**Yang**	2024	Korea	Korea National Health Insurance Service cohort	209,424	17 potential predictors, e.g., pregestational and postpartum depression, anxiety, cesarean delivery, social economic status	Prenatal and postnatal	Motor and cognitive developmental disorders	5–14y	RFM, LR	None	The highest importance values for predicting neurodevelopmental delay were found for low socioeconomic status and mother’s age at birth. While RFM yielded a relatively high AUC of 0.74, logistic regression yielded a low AUC value of only 0.50.	10/12
**Zhou**	2024	China	Peking University Birth Cohort in Tongzhou	1,125	17 sociodemographic, behavioral, and medication-usage variables, and chemical exposure	Prenatal	Symptoms of neurodevelopmental disorders using ASQ-3	12m	ANNs, RFM, SVM, LR	LASSO	Certain maternal exposures (acetaminophen, ferrous succinate, midazolam) were associated with higher risks of neurodevelopmental abnormalities during the first year. ANNs achieved good performance in predicting overall neurodevelopmental issues (AUC = 0.821).	8/12

Abbreviations: ACARS, Autism: Childhood Autism Rating Scale; ADHD, Attention Deficit/Hyperactivity Disorders; ANN, Artificial Neural Network; ANOVA, Analysis of Variance; APGAR, the Appearance, Pulse, Grimace, Activity, and Respiration score; ASD, Autistic Spectrum Disorder; ASQ, Ages and Stages Questionnaire; BAS, the British Ability Scales Early Years Battery, 2^nd^ Edition; BDI, Battelle’s Developmental Inventory; BITSEA, Brief Infant-Toddler Social Emotional Assessment;BSID-II, Bayley Scales of Infant Development – Second Edition; CBCL, Child Behavior Checklist; DAS-II, Differential Ability Scales – Second Edition; DT, Decision Tree; FASD, Fetal Alcohol Spectrum Disorders; GDS, the Chinese version of the Gesell Development Scale; GEC, Global Executive Composite; ICD 10, International Classification of Diseases; IED, Intra-Extra Dimensional Shift Set; INFANIB, Infant Neurological International Battery;; KBIT-2 IQ, Kaufman Brief Intelligence Test Second Edition; LR, Logistic Regression; MDI, Mental Developmental Index; PCA, Principal Component Analysis; PDI, Psychomotor Development; RFM, Random Forest Model; ROC, Receiver Operating Characteristic; SVM, Support Vector Machines; VABS, Vineland Adaptive Behavior Scales; WISC-IV, Wechsler Intelligence Scale for Children – Fourth Edition.

1 y: years old, m: months old, w: weeks old, and ± followed by standard deviation.

2 This column includes statistical approaches based on computing a closed-form expression that does not require iteration or learning processes for determining model parameters.

3 This article reports detailed results only on the RFM, while mentioning that SVM and LR models were trained as well.

**Table 3:** Characteristics of pattern-focused ML studies.

First author	Year	Country	Data source	Sample size	Maternal exposure to risk factors/ predictors	Timing of exposure	Child outcome assessment	Age of assessment^1^	Main machine learning technique and/or other statistical approach	Main findings
**Cao**	2024	China	Wuhan birth cohort	809	Rare earth elements	Prenatal	Neurodevelopment (MDI, PDI)	2 y	BKMR, GEE	Higher rare earth element levels in the first and third trimesters were associated with lower MDI and PDI scores, with thulium, erbium, cerium, and lanthanum having the greatest impact.
**Chen**	2023	China	Shanghai-Minhang birth cohort	424	Bisphenol A	Prenatal	WISC-IV	6 y	BKMR, QGC, GAM, multiple linear regression	Prenatal exposure to bisphenol was linked to lower IQ in boys in a non-linear pattern.
**Chiu**	2023	USA	ACCESS pregnancy cohort	236	Ambient air pollutant mixture	Prenatal	WRAML-2, CPT-II memory-related and executive functions	6.5 y	BKMR-DLM, WQS	Ambient air pollutant exposure was associated with decreased general memory and memory-related attention and concentration.
**Coker**	2017	USA	CHAMACOS Cohort	255	Exposure to 15 potentially neurotoxic pesticides	1st, 2nd, and 3rd trimesters	FSIQ assessed using WISC-IV	7 y	BKMR, BPR with MCMC estimation	BPR identified 8 clusters of pesticide profiles. Profiles associated with high pesticide use were also associated with deficits in adjusted FSIQ of up to -6.9.
**Conejo-Bolaños**	2024	Costa Rica	Community-based birth cohort	355	Pesticide	Prenatal	Neurodevelopmental outcomes using BSID-III	12 m	BKMR	Higher prenatal commonly used pesticide levels were linked to lower language and motor scores in all children. Increased chlorpyrifos exposure was associated with lower cognitive scores overall and reduced motor scores in boys but not girls. Higher pyrimethanil levels were tied to lower language abilities in girls. Pyrethroid metabolites showed no impact, and no non-linear or mixture effects were found.
**Enright**	2023	USA	CIOB and IKIDS cohorts	163	Per- and polyfluoroalkyl substances	Prenatal	Visual recognition memory (measured using eye-tracking paradigms)	7.5 m	BKMR, GAM	Higher PFAS levels, including PFNA, PFOA, PFOS, PFHxS, PFDeA, and PFUdA, were linked to an increased shift rate. Similarly, higher PFAS mixture levels showed a modest positive association with shift rate. However, PFAS exposure was not significantly related to familiarization time, processing speed, or visual recognition memory.
**Fruh**	2021	USA	Project Viva	1,009	Exposure to lead, manganese, selenium, and methylmercury	Prenatal (2nd trimester)	BRIEF, GEC, SDQ	6-11y	BKMR, multivariable linear regression and quantile g-computation	The authors did not observe strong evidence of adverse effects of the mixture of targeted metals (lead, manganese, mercury, and selenium) on neurobehavior.
**Gao**	2020	China	Sheyang Mini Birth Cohort Study	326	Exposure to heavy metals, pesticide metabolites, and phenols	Prenatal	C-WISC	7y	BKMR, ENR	Prenatal exposure to selected chemical mixtures may affect intellectual performance at 7 years of age, particularly in boys. Lead and bisphenol A were suspected as primary chemicals associated with child neurodevelopment.
**Goodrich**	2024	USA	Childhood Autism Risk from Genetics and the Environment (CHARGE) population-based case-control study	1,413	Exposure a mixture of air pollutants, including ozone and ultrafine particulate matter	Pre-pregnancy, prenatal, and postnatal (2y)	VABS, MSEL	47.2 m (range 38.5–53.9 m) for ASK, 45.5 m (range 36.8–53.0 m) for non-ASD	(Hierarchical) BKMR	Strong negative associations with all scores were reported for pre-pregnancy ozone. Weak and inconsistent associations were observed for ozone during year 1 and strong negative associations for particulate matter were observed during year 2.
**Gu**	2024	China	Guangxi Zhuang Birth Cohort	211	Exposure to 17 metals	Prenatal (1st trimester)	C-WISC	3-6 y	BKMR, GLM, QGC, RCS, WQS	IQ scores declined as non-essential metal concentrations increased, even after adjusting for essential metals. Non-essential metals included arsenic, rubidium, strontium, cadmium, antimony, cesium, barium, tungsten, lead, and uranium. Among them, uranium had the strongest negative impact, followed by lead and antimony.
**Hernandez-Castro**	2023	USA	MADRES population-based cohort	204	Exposure to pesticides	Prenatal	CBCL	3 y	BKMR	Maternal organophosphate exposure was linked to behavioral problems in children, with sex-specific effects for some compounds.
**Huang**	2007*	Seychelles	Seychelles Child Development Study	643	Methylmercury exposure (from fish consumption)	Prenatal	WISC III FSIQ	9 y	Regression tree	Exposure to methylmercury was measured using the concentration in maternal hair that had grown during pregnancy. The authors found directionally opposing effects of MeHg exposure among children with different backgrounds and IQ levels.
**Kalloo**	2021	USA	Cohort subset (Health Outcomes and Measures of the Environment Study)	253	Chemical exposure, 43 metals, phthalates, phenols, polybrominated diphenyl ethers	Prenatal (at 16 and 26 gestational weeks)	WISC-III, WISC-IV	5 & 8y	k-means clustering and principal component analysis	The authors reported that children born to women with profiles of gestational chemical exposure characterized by higher biomarker concentrations of Arsenic, Cadmium, Mercury, most phthalates, phenols, organochlorine compounds, and organophosphate pesticides had lower cognitive ability scores at ages 5 and 8 years.
**Kou**	2025	Spain	A subset of ECLIPSES cohort	400	Exposure to heavy metals	Prenatal	BSID-III	40 days	BKMR, GAM, RCS, WQS, multivariable linear regression	Prenatal exposure to cadmium and nickel was linked to delays in infant language development, with cadmium also affecting cognitive development. Lead showed a non-linear association with language. The metal mixture had a significant adverse effect on expressive language.
**LaLonde**	2018	Seychelles	Seychelles Child Development Study	533	Methylmercury exposure (from fish consumption)	Prenatal	CBCL, CTRS, CVLT, KBIT, WISC-III, and other 8 motor tasks, such as finger tapping, etc.	9 y	Bayesian multiple outcomes model with DPMM and MCMC	The authors showed that there was no statistically significant association between prenatal MeHg exposure and cognitive, motor, and behavioral development.
**Lee**	2021	South Korea	Cohort subset (Environment and Development of Children Study)	502	Metals mixture exposure	Prenatal (2nd trimester); Study also included data from postnatal exposure at ages 4 and 6 since participation rate was high.	KEDI-WISC	6y	BKMR, quantile g-computation models, and elastic net models	The authors reported that there were statistically significant inverse associations between prenatal and postnatal level of the four metals and total IQ scores at the age of 6 years in the multi-chemical models. Moreover, manganese level at both age 4 and 6 years was the strongest contributing factors to children’s IQ.
**Li**	2020	China	Wuhan Women and Children Medical Care Center	504	Metals exposure (strontium, selenium and manganese) in prenatal (urine sample)	Prenatal	Bailey II (MDI + PDI)	2y	BKMR, linear regression	The results shown in girls revealed that MDI scores were improved with increasing concentrations of strontium, selenium and manganese mixture until the concentrations reached their 30^th^ percentiles, with no effect after that threshold level. No signification association of mixture with MDI/PDI was found in boys.
**Li**	2024	China	Ma’anshan birth cohort	2,410	Exposure to pesticides	Prenatal	ADHD symptoms using Conners Abbreviated Symptom Question	3, 5, and 6 y	BKMR, GLM, QGC, RCS, multinomial logistic regression	Prenatal exposure to dibutyl phosphate, bis(2-butoxyethyl) phosphate, and diphenyl phosphate was linked to increased risk in the high-score ADHD group. Bis(2-butoxyethyl) phosphate in the third trimester was associated with a decreased risk in the moderate-score group.
**Li**	2025	China	Tianjin Maternal and Child Health Education and Service cohort	172	Exposure to polycyclic aromatic hydrocarbons	Prenatal	Neurodevelopment using GDS (Chinese version)	6-12 m	BKMR, WQS, multivariable linear regression	Exposure to polycyclic aromatic hydrocarbons during early pregnancy was linked to negative impacts on child neurodevelopment, particularly in personal social and language scores.
**Liu**	2018	Mexico	The PROGRESS, a prospective birth cohort	665	Exposures to a panel of nine metals (arsenic, cadmium, cobalt, chromium, cesium, copper, manganese, lead, and antimony)	2^nd^ trimester of pregnancy	BSID-III composite cognition scores	6, 12, 18, and 24m	BVCKMR	The authors reported that the positive association between copper exposure and neurodevelopmental outcomes at 24 months was diminished in the presence of elevated lead levels. An interaction effect between second trimester copper and lead exposures for cognition at 24 months was also observed.
**Liu**	2022a	China	Cohort subset (Guangxi Birth Cohort)	703	Metals exposure (22 targeted metals)	Prenatal (1st, 2nd, and 3rd trimesters)	GDS	2-3y	BKMR, ENR, linear regression	Prenatal aluminum exposure was negatively associated with the fine motor developmental quotient, adaption, language, and social development. Prenatal cadmium exposure was negatively associated with gross motor, while postpartum cadmium exposure was negatively associated with language. The first and second trimester might be the most sensitive period when metal exposure affects neurodevelopment.
**Liu**	2022b	China	Cohort study (Longhua Child Cohort)	26,052	Multiple ambient air pollutants	Prenatal (the 7^th^ month pregnancy) and postnatal (the 4^th^ month after birth)	Conners’ Hyperactive Index, ADHD, hyperactivity index	3.5y	DLNM, RFM**	Early life exposure to PM10, PM2.5 and NO2 was associated with an increased risk of child ADHD-like behaviors at the age around 3 years, and the late-prenatal and early postnatal periods might be the susceptible exposure windows.
**Long**	2024	China	Guangxi Zhuang birth cohort	221	Alkylphenols	Prenatal	WPPSI-IV	3-6y	BKMR, GLM, RCS	Prenatal exposure to certain alkylphenols, such as nonylphenol and 4-T-OP, was associated with lower IQ and working memory scores in children, while exposure to 4-N-NP showed a positive association with fluid reasoning, especially in girls.
**Ni**	2024	USA	CANDLE and TIDES combined cohort	814	Polycyclic aromatic hydrocarbons	Prenatal	Hearts and Flowers Task for cognitive flexibility, Digital Span subset (WISC) for working memory, and NIH Toolbox Flanker Inhibitory Control and Attention Test	8-9y	BKMR, linear regression	The primary analysis of linear regressions showed generally null results, with no significant modification by child sex or maternal stress. Mixture analyses indicated several pairwise interactions between PAH metabolites affecting working memory, particularly between 2/3/9-FLUO and other metabolites, but no overall or individual effects were found.
**Oskar**	2024	USA	High risk cohort	130	Phenol, paraben, and pesticide	Prenatal	MSEL	3y	MFVB-LKMR	Phenol, paraben, and organophosphate insecticide exposure in the second and third trimesters was linked to differences in 3-year neurodevelopmental outcomes, with evidence of trimester- and sex-specific effects, particularly among females.
**Oulhote**	2019	Faroe Islands	National Hospital in Tórshavn	465	Exposure to mercury and six other persistent organic pollutants (that accumulate in marine food sources)	Prenatal at 32 weeks of pregnancy	BNT and SDQ	7 y	Super Learner (ensemble ML technique incorporating GLM, GAM, ENET, MAPSR, SVM, RFM, GB, ANN), G-computation	Maternal Hg, perfluorooctanoic acid (PFOA), and perfluorooctane sulfonic acid (PFOS) exposure was associated negatively with BNT scores. Regarding SDQ scores, PFOS and PFOA were positively associated with higher (poorer) scores, while polychlorinated biphenyls (PCBs) were negatively associated.
**Qui**	2024	China	Jiangsu birth cohort	854	Metal	Prenatal	Neurodevelopment using Bayley-III	3y	BKMR, RCS multivariable linear regression, Poisson regression	Prenatal exposure to manganese was linked to a lower risk of non-optimal cognition development, while exposure to vanadium, copper, zinc, antimony, cerium, and uranium increased the risk of non-optimal gross motor development. A risk score based on multiple metal exposures identified a higher probability of non-optimal gross motor development in children with the highest exposure levels, with antimony and uranium being the main contributors.
**Rokoff**	2022	USA	Cohort data (New Bedford Cohort)	468	Exposure to mixture of organochlorines and metals	Prenatal	The Conners’ Rating Scale (CRS, at 8y); the Behavior Assessment System for Children, Second Edition (BASC-2, at 15y)	8y & 15y	BKMR and five-chemical linear regression models	The authors reported the overall mixture was positively associated with Conners’ Parent Rating Scale and Self Report of Personality anxiety and depressive symptoms, and negatively with somatic symptoms. Prenatal lead was positively associated with adolescent anxiety symptoms and a doubling of cord blood manganese was positively associated with internalizing symptoms for girls, but not boys.
**Shin**	2022	South Korea	Cohort study (Mothers and Children’s Environmental Health Study)	353	Multiple ambient air pollutants	Prenatal and postnatal	The Korean version of the Child Behavior Checklist	5y	BKMR, multivariate linear regression	The authors reported that the effects of multiple air pollutants during the first trimester of pregnancy and 0–6 months of the infantile period were significantly associated with behavioral problems. Boys showed a stronger association than girls.
**Song**	2023	USA	Early Autism Risk Longitudinal Investigation cohort	154	A mixture of persistent pollutants	Prenatal	MSEL-ELC, SRS, VABS	3y	BKMR, QGC, linear regression	Higher levels of a flame-retardant chemical were linked to more social difficulties, while higher levels of a pesticide were associated with fewer social challenges. Two industrial chemicals were connected to better cognitive skills, and several pollutants were linked to improved adaptive functioning. No overall effect of pollutant mixtures was found, but some flame retardants may interact with each other.
**Surabhi Shah-Kulkarni**	2020	South Korea	Cohort data (Mothers and Children’s Environmental Health Study)	523	Exposure to lead, mercury, and cadmium	Prenatal (early pregnancy 12–20 weeks & late pregnancy >28 weeks)	KBSID-II (MDI + PDI)	6m	BKMR, GAM	Combined exposure to lead and mercury during late pregnancy affects neurodevelopment in infants at 6 months. Exposure to lead in late pregnancy was found to result in most neuro-toxic effects.
**Valeri**	2017	Bangladesh	Cohort data (clinics operated by the Dhaka Community Hospital Trust)	825	Exposure to metal mixture, arsenic, manganese, and lead	Prenatal	Bayley III	20-40m	BKMR and multivariate regression	The authors reported that co-exposure to arsenic, manganese, and lead during gestation was jointly neurotoxic and affected neurodevelopment of Bangladeshi children in early life stages in a complex fashion. Manganese at high concentrations was found to be the most neuro-toxic component of the metal mixture.
**Vuong**	2020	USA	Cohort subset (Health Outcomes and Measures of the Environment Study)	161	Persistent organic pollutants including 5 polybrominated diphenyl ethers, 6 polychlorinated biphenyls, dichlorobiphenyls, dichloroethylene, dichlorodiphenyltrichloroethane, and 4 polyfluoroalkyl substances	Prenatal	Reading skills: Wide Range Achievement Test-4 reading composite score	8y	BKMR, BART, ENET, LASSO, SPCA, WQS	The authors reported inverse associations between prenatal polybrominated diphenyl ethers concentrations and children’s reading scores. Positive associations of polychlorinated biphenyls congeners and polyfluoroalkyl substances with reading skills were also found.
**Wang**	2024	China	Guangxi Zhuang birth cohort	4,494	Fine particulate matter	Prenatal	Neurodevelopmental disorders and behavior problems using the Warning Signs Checklist	3, 6, 8, 12, 18, 24, 30, 36 months & 4, 5, 6 years	BKMR, WQS, GAM, Poisson regression	Prenatal exposure to fine particulate matter increased the risk of neurodevelopmental and behavioral problems, with stronger effects in early pregnancy. Among PM2.5 components, sulfate posed the highest risk, while organic matter contributed the most to overall impact. These findings highlight the need to reduce PM2.5 exposure during pregnancy to lower neurodevelopmental disorder risks in children.
**Wei**	2023	China	Cohort data (Nanjing Maternity and Child Health Care Hospital)	710	Pesticides	Prenatal	ASQ	12 & 18m	BKMR, RCS, GAM, WQS	The authors found significant inverse associations between prenatal exposure to chlorpyrifos, mirex, atrazine, dimethipin and the domain-specific neuropsychological development (i.e., communication, gross motor and fine motor) of children at 12 and 18 months of age.
**Xie**	2022	China	Cohort data (Shanghai-Minhang Birth Cohort Study)	614	Perfluoroalkyl	Prenatal (12–16 gestational weeks)	CBCL 1.5–5	2 & 4y	BKMR	The authors found no clear evidence that prenatal exposure to Perfluoroalkyl substances had negative effects on neurobehavioral development in children.
**Xu**	2022	China	Cohort data (Jiangsu Birth Cohort Study)	1,531	PM2.5 exposure	Prenatal (8–14 gestational weeks)	Bayley III	12m	Multivariable Poisson regression with generalized linear mixed models	Results suggested that prenatal exposure to PM_2.5_, particularly with high SO4^2-^ concentration, was associated with children’s non-optimal gross motor development at 1 year old.
**Yang**	2024	China	Shanghai birth cohort	1,765	Per-and polyfluoroalkyl substances	Prenatal	BRIEF-P	4y	BKMR, RCS, multivariable linear regression	The authors reported no significant associations between prenatal PFAS exposure and BRIEF-P scores when the child was 4 years old.
**Yim**	2022	Japan	Cohort data (Sapporo cohort, Hokkaido Study on Environment and Children’s Health)	259	Exposure to dioxins and polychlorinated biphenyls	Prenatal	Bayley II	6m	BKMR and quantile-based g-computation	The authors reported that there was little evidence of inverse associations of prenatal exposure to dioxins and polychlorinated biphenyls with infant psychomotor development.
**Yonkman**	2023	Canada	The Maternal-Infant Research on Environmental Chemicals (MIREC) study cohort	517	Gestational chemical mixtures: 27 potential neurotoxicants (1^st^ trimester); 5 metals, 4 organochlorine pesticides, 5 organophosphate pesticides metabolites, 4 phthalate metabolites plus the molar sum of Bis(2-ethylhexyl) phthalate (DEHP) (comprised of mono-[2-ethyl-5-hydroxyhexyl] phthalate, mono-[2-ethylhexyl] phthalate, and mono-[2-ethyl-5-oxohexyl] phthalate), 6 polychlori- nated biphenyls (PCBs), 1 polybrominated diphenyl ether (PBDE), and cotinine	1^st^ trimester	WPPSI-III	3.4 y (range 2.8–4.2 y)	Latent profile analysis, k-means	The authors observed negative associations between both the smoking chemicals and high monoethyl phthalate profiles and all IQ scores and between the high persistent organic pollutants (POP) profile and Full-Scale and Verbal IQ scores. Moreover, a positive association between the low POP profile and Full-Scale and Performance IQ scores was also reported.
**Zhang**	2023	China	Cohort data (Sheyang Mini Birth Cohort Study)	716	Exposure to perfluoroalkyl	Prenatal	DST at 1 year old and GDS at 2 & 3 years old	1, 2, & 3y	BKMR, GEE, GLM	Prenatal perfluoroalkyl substances exposure may be associated with adverse neurodevelopment effects in the first 3 years of life.
**Zhang**	2024	China	Sheyang Mini birth cohort	327	Per- and polyfluoroalkyl substances	Prenatal	Intelligence quotient using WISC-CR	7y	BKMR, GLM	Perfluorinated carboxylic acid concentration in cord serum was negatively associated with performance IQ of 7-year olds.
Zhou, T***	2025	South Africa	Drakenstein Child Health study	545	Phenols	Prenatal	Bayley III	2y	BKMR, SOM, QGC, WQS, linear regression	Overall, the authors reported no significant association between phenol exposure and cognitive neurodevelopment. For specific subgroups, however, the authors found that higher pentachlorophenol (PCP) concentrations were associated with lower cognitive scores among non-smokers, higher bisphenol A (BPA) concentrations were associated with lower cognitive scores among males, and higher bisphenol S (BPS) concentrations were associated with lower cognitive scores in those with moderate-high socioeconomic status.
Zhou, J.****	2025	China	Ma’anshan birth cohort	1,586	Neurotoxic metals	Prenatal	WPPSI-IV	3-6y (preschool)	BKMR, QGC, RCS, multiple linear regression, interaction and marginal effects models	Higher placental arsenic, cadmium, and mercury concentrations were associated with lower cognitive scores (e.g., FSIQ, VCI, VSI, and FRI) at preschool age. These negative associations were observed only in boys after stratifying by sex.

Abbreviations: ACCESS, the Asthma Coalition on Community, Environment and Social Stress project; ADHD, Attention Deficit/Hyperactivity Disorders; ANN, Artificial Neural Network; ASQ, Ages and Stages Questionnaire; BART, Bayesian Additive Regression Trees; Bayley III, Bayley Scales of Infant Development, Third Edition; BKMR, Bayesian Kern Machine Regression;; BPR, Bayesian Profile Regression; BNT, Boston Naming Test; BRIEF, Behavior Rating Inventory of Executive Function; BRIEF-P, Behavior Rating Inventory of Executive Function-Preschool version; BSID-II/III, Bayley Scales of Infant Development, Second/Third Edition; BVCKMR, Bayesian Varying Coefficient Kernel Machine Regression; CANDLE, Conditions Affecting Neurocognitive Development and Learning in Early Childhood cohort; CBCL, Child Behavior Checklist; CIOB, the Chemicals in Our Bodies cohort; C-MACG, the Chiba Study of Mother and Child Health; CPRS, Conners’ Parent Rating Scales; CPT-II, Conners’ Continuous Performance Test; CTRS, Conners’ Teacher Rating Scale hyperactivity index; CVLT, California Verbal Learning Test; C-WISC, Chinese version of Wechsler Intelligence Scale for Children; DLM, Distributed Lag Models; DLNM, Distributed Lag Non-linear Models; DST, Developmental Screen Test for Children; ENET, Elastic Net; ENR: Elastic Net Regression; FSIQ, Full Scale Intelligence Quotient; GB, Gradient Boosting; GAM, Generalized Additive Models; GDS, Chinese version of the Gesell Development Scale; GEC, Global Executive Composite; GEE, Generalized Estimating Equations; GLM, Generalized Linear Regression; GUSTO, Growing Up in Singapore towards Healthy Outcomes birth cohort; INMA, INfancia y Medio Ambiente Study; IKIDS, Illinois Kids Development Study cohort; KBIT IQ, Kaufman Brief Intelligence Test; KBSID-II, Korean version of Bayley Scales of Infant Development II; KEDI-WISC, Korean Educational Developmental Institute’s Wechsler Intelligence Scale for Children; LASSO, Least Absolute Shrinkage and Selection Operator; MADRES, Maternal And Developmental Risks from Environmental and Social Stressors study cohort; MAPSR, Multivariate Adaptive Polynomial Spline Regression; MCHAT, the Modified Checklist for Toddlers with Autism; MCMC, Markov Chain Monte Carlo; MDI, Mental Developmental Index; MFVB-LKMR, Mean Field Variational Bayes for Lagged Kernel Machine Regression; MSCA, McCarthy Scales of Children’s Ability; MSEL, Mullen Scales of Early Learning; PDI, Psychomotor Development Index; QGC, Quantile g-computation; RCS, Restricted Cubic Splines; RFM, Random Forest Model; SDQ, the Strengths and Difficulties Questionnaire; SEPAGES, French Assessment of Air Pollution exposure during Pregnancy and Effect on Health cohort; SES, socioeconomic status; SOM, Self-organizing Maps; SPCA, Sparse Principal Component Analysis; SRS, Social Responsiveness Scale; SVM, Support Vector Machine; TIDES, Infant Development and the Environment Study cohort; VABS, Vineland Adaptive Behavior Scales; WISC (-III/IV), Wechsler Intelligence Scale for Children (3^rd^ edition/4^th^ edition); WPPSI-III/IV, the Wechsler Preschool and Primary Scale of Intelligence, Third Edition/Fourth Edition; WQS, Weighted Quantile Sum; WRAML-2, Wide Range Assessment of Memory and Learning. * A follow-up paper [68] to Huang 2007 was not included in this scoping review, as their outcome variables are measured in late adolescence/early adulthood. ** The authors used REM to evaluate level of exposure level of each subject. For the purpose of the current review, this is a method as an input variable but not as an outcome variable. *** Zhou, T represents study “Zhou, T., Abrishamcar, S., Christensen, G., Eick, S. M., Barr, D. B., Vanker, A.,... & Hüls, A. (2025). Associations between prenatal exposure to environmental phenols and child neurodevelopment at two years of age in a South African birth cohort. *Environmental Research*, *264*, 120325.” **** Zhou, J represents study “Zhou, J., Tong, J., Liang, C., Wu, P., Ouyang, J., Cai, W.,... & Huang, K. (2025). Prenatal metals and offspring cognitive development: Insights from a large-scale placental bioassay study. *Environmental Research*, *267*, 120684.“

1 y: years old, m: months old, w: weeks old, and ± followed by standard deviation.

For studies employing ML techniques to study only the association between variables without an objective to predict outcomes (pattern-focused ML studies), the quality assessment tool used in the current review is not applicable. Among the 44 studies in this subgroup, listed in Table 3, the majority examined populations from China (17 studies) and the USA (10 studies), followed by South Korea (3 studies), and the Seychelles (2 studies). The remaining studies were conducted in the Faroe Islands, Costa Rica, Spain, Mexico, Bangladesh, Japan, Canada, and South Africa, each represented by one study. These studies examined a range of developmental outcomes, with neurodevelopment, including motor development, accounting for the largest proportion at 36%, followed by cognitive development at 31%. Behavior and social-emotional development accounted for 19%, while neurodevelopmental disorders represent the smallest share at 14%. This distribution highlights the varying emphasis on different aspects of child development (S2 Fig).

### Two main objectives of machine learning techniques in studying maternal exposure to risk factors and child outcomes

From the analysis of the 60 studies in this review, two distinct subgroups were identified based on their objectives for employing ML techniques in constructing statistical models for child development outcome variables including ADHD, ASD, fetal alcohol spectrum disorders (FASD), and developmental problems. The first subgroup, comprising 16 studies, prioritizes predictive diagnostic applications. These prediction-focused ML studies are characterized by a diversity of risk factors and methodological approaches. The second subgroup, which comprises the majority (44 studies, 73.3%), aims to infer relationships between risk factors and child development outcome variables. A predominant focus of this subgroup of pattern-focused ML studies is on external risk factors, such as exposure to air pollutants [69–71], organic pollutants [72,73], chemical mixtures [58,74–80], metals [74,76,81–89], pesticides [74,83,90,91], methylmercury [82,92,93], neurotoxicants [74], or alcohol [55,56,94–96]. Additionally, some studies emphasized internal risk factors (or covariates), such as mental and nervous system disorders during pregnancy [97], birth conditions [98], psychological symptoms [96,66,67,99,100], psychosocial stressors, gestational diabetes, or other perinatal complications [58,59,70,73,77,87,91].

In the subgroup of prediction-focused ML studies (Table 2), six studies focused on predictors mainly during the prenatal period, six included both the pregnancy and postnatal periods, and four considered only the postnatal period. A wide range of risk factors were included across these studies, with alcohol exposure highlighted in five studies [55,56,94–96] and smoking during pregnancy in two [55,94]. Maternal depression or psychopathological symptoms were assessed in six studies [94,96,66,67,99,100], while one study focused on perinatal factors such as diseases during pregnancy, mental and nervous system disorders, and complications like intrapartum hemorrhage [97]. Exposure to cytokines was investigated in one study [57], and bio-physiological predictors, including maternal data and milk chemical composition, were included in two [75,98]. Toxic exposures, such as paints, solvents, and heavy metals, were reported in one study [58], while biological hazards spanning pre-, peri-, and post-natal periods were covered in another [59]. The authors reported moderate to good accuracy for prediction models for ADHD [56], and FASD (1^st^ year postpartum) [55], and diagnosis of ASD [57,58,97,98]. In two studies aiming to predict ASD in children aged 4 years or older, one study based on register data yielded low accuracy and no improvement after adding data from maternal inflammatory markers from early pregnancy [57]. In two large cohort studies focusing on early ASD symptoms and ASD diagnosis in children aged 2 years or older, depressive symptoms were not included in the final feature importance results [66,67]. The other study, based on several biomarkers such as maternal familial history of auto-immune diseases, reported moderate accuracy [98]. In the context of predicting developmental problems, Li et al. [75] and Soleimani et al. [59] reported results with moderate accuracy based on milk samples and biological problems (e.g., high-risk pregnancy, respiratory distress syndrome, etc.), respectively. Bowe et al. [95] reported that prenatal exposure to an air pollution mixture was linked to lower general memory and attention/concentration scores in children aged 5 years, suggesting poorer memory function. Furthermore, based on maternal psychological symptoms, Usta & Karabekiroğlu [99] reported moderate accuracy in predicting children’s social-emotional problems between 12 and 42 months of age. Finally, alcohol consumption, along with various maternal socioeconomic factors, was found to predict the likelihood of a child having an IQ score below 90 at 5 years old [94].

In the subgroup of pattern-focused ML studies (Table 3), five studies investigated risk factors from multiple ambient air pollutants or fine particulate matter (PM_2.5_) [69–71,101,102], while other 39 studies focused on various chemical or metal exposures. Regarding the timing of exposure, only four studies included both prenatal and postnatal phases. The other 40 studies investigated exposure only during the prenatal phase. Among these 40 studies, six reported that there is little to no evidence to support the impact of exposure to external risk factors (e.g., phenols), during pregnancy on neurodevelopment or psychomotor development [77,78,82,93,103,104]. The other 33 studies reported significant associations, showing a negative impact on cognitive development [74,76,84,86,90,92,101,105–111], neurodevelopment [69,74,79,83,88,89,91,102,112–115], behavior problems [70,102,116], motor development [71,117,118], word retrieval, language development [72,117,119], and reading skills [73]. Interestingly, one study reported that second-trimester copper exposure was positively associated with cognitive development at 24 months and cognitive trajectories from 6–24 months, with an interaction effect between copper and lead exposures [81]. In addition, Qiu et al. reported prenatal exposure to manganese was linked to a lower risk of non-optimal cognition development, suggesting a protective effect [118]. Another study reported that higher levels of a flame-retardant chemical were linked to more social difficulties, while higher levels of a pesticide were associated with fewer social challenges. Two industrial chemicals were connected to better cognitive skills, and several pollutants were linked to improved adaptive functioning [120]. The timing of exposure, targeted predictors, outcome assessment tools, and age range of the children all differed from one study to another.

### Type of machine learning techniques used

In the subgroup of prediction-focused ML studies, the procedure of splitting into train, test, and validation data as well as corresponding performance metrics were typically reported. By contrast, in the subgroup of pattern-focused ML studies, ML techniques were employed to describe relationships in complex data that can be challenging to analyze with traditional methods. Given the studies in this subgroup did not provide predictions, validation techniques for prediction performance were typically not employed and issues such as overfitting were not investigated.

**Fig 2:**
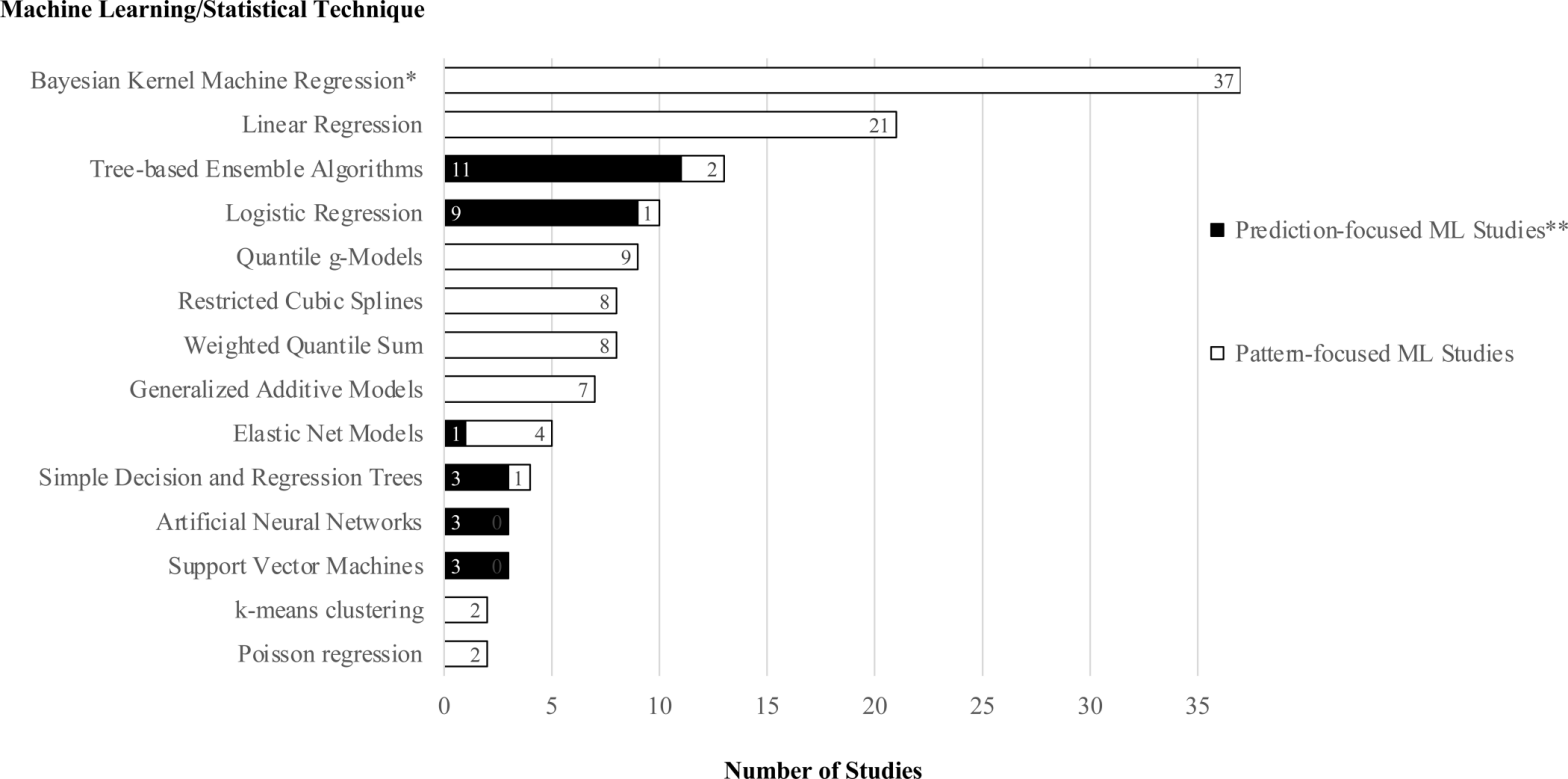
Overview of the frequency of machine learning and statistical techniques used in the included studies. This figure displays the various techniques employed across the included studies. On the left, the specific techniques are listed, while the bars represent their frequency of use. The following techniques were each employed in only one of the included studies: Bayesian additive regression trees, Bayesian multiple outcomes model, Bayesian profile regression, distributed lag non-linear models, generalized linear mixed models, latent profile analysis, least absolute shrinkage and selection operator, principal component analysis, Super Learner (ensemble machine learning). *Includes variants of the technique such as Bayesian varying coefficient kernel machine regression. ** For prediction-focused ML studies, only the machine learning/statistical technique in the main part of the analysis but not those used for robustness checks were included in the count.

An overview of the methodological characteristics of the prediction-focused ML studies is provided in Table 2 and Fig 2. In these studies, the most frequently used techniques were Decision Tree Models, Logistic Regressions, and Artificial Neural Networks (ANNs). Eleven of the fourteen decision tree models were tree-based ensemble algorithms such as Random Forest Models (RFMs) [55,57,75,80,94,95,100] and Gradient Boosting Decision Trees [66,67,97,98]. The remaining three models were simple single decision trees [56,96,99]. The three ANNs were all relatively small fully connected feed forward ANNs. In the first study by Grossi et al., the ANN had an input layer of 16 nodes, one hidden layer of 12 nodes and an output layer of two nodes [58]. In the second study by Soleimani et al., the ANN had an input layer of 14 nodes, one hidden layer with also 14 nodes, and an output layer with one node [59]. In the third study by Zhou et al, the final ANNs had an input layer of five nodes, one hidden layer of four nodes, and a single output node [80]. Consistent with typically small sample sizes, model size and complexity were relatively small in all of these studies. To put this into the context of general recommendations around sample size of ANNs, for instance, Alwosheel et al. [121] recommended, a sample size of 50–1,000 times the number of model weights. Thus, even for the relatively small networks in the ANN studies reported between 1,000 and 20,000 independent observations would have seemed appropriate.

In the subgroup of pattern-focused ML studies, the most frequently used technique is Bayesian Kernel Machine Regression (BKMR). This statistical approach was originally introduced to the estimation of health effects of pollutant mixtures by Bobb et al. [122]. It combines Bayesian inference and kernel-based ML to model complex, non-linear relationships in high-dimensional data, yielding interpretable outcomes. BKMRs were employed by 36 out of 44 studies in this subgroup. Of the other eight studies, two further studies employed methods related to BKMR such as Bayesian Varying Coefficient Kernel Machine Regression (BVCKMR) [81] Mean Field Variational Bayes for Lagged Kernel Machine Regression (MFVB-LKMR) [115]. Two studies employed clustering algorithms in combination with traditional regression methods [74,76]. In this context, clustering served the purpose of handling the complexity and high dimensionality of the data before applying simpler methods that are easier to interpret. One study employed Distributed Lag Non-linear Models (DLMNs) for the main part of the analysis, while in a pre-step to this analysis, an RFM was used to estimate the input variable chemical exposure level [69]. Another study employed a Multivariable Poisson Regression (MPR) with Generalized Linear Mixed Models (GLMMs), while again, in a pre-step to the analysis, chemical exposure levels were estimated by an RFM [71].

The level of methodological maturity appears to differ substantially between the two types of studies. In the prediction-focused ML studies, a high degree of heterogeneity of algorithms and procedures indicates that predictive applications of ML are still in a phase of methodological experimentation. Among these studies, it is therefore difficult to identify a clear best-practice example. Soleimani et al. [59], for instance, provide a well-documented example of how to construct, document, and evaluate a simple ANN. However, this method has yet to gain traction and is constrained by limitations in transparency and ability to predict outcomes with small datasets. By contrast, a commendable example of accomplishing transparency and using a method appropriate for their sample size was provided by Goh et al. [56]. They utilized a simple decision tree that was presented in its entirety within their article and validated its performance with an independent sample. Despite its strengths, such as transparency and scalability to smaller datasets, this approach would struggle to handle larger, more complex datasets due to its limited expressiveness and vulnerability to overfitting. In these scenarios, more sophisticated methods such as RFMs, as employed in other studies [55,57,75], could offer a solution. However, these studies fell short in terms of interpretability and failed to consistently apply rigorous performance validation.

In contrast to the prediction-focused ML studies, the pattern-focused ML studies showed a certain convergence to a standard methodology. In this context, ML techniques coalesced into a pre-defined template or recipe to address the dimensionality and complexity of data. As an example, Valeri et al. [88] provided a BKMR template for handling complex and multi-dimensional data that was used successfully by several later studies. As mentioned earlier, BKMR works by combining Bayesian hierarchical modeling with kernel methods. This method is particularly well-suited for situations where the relationships between variables are nonlinear and interactive, such as when modelling environmental exposure mixtures.

### Overall quality assessment

**Fig 3:**
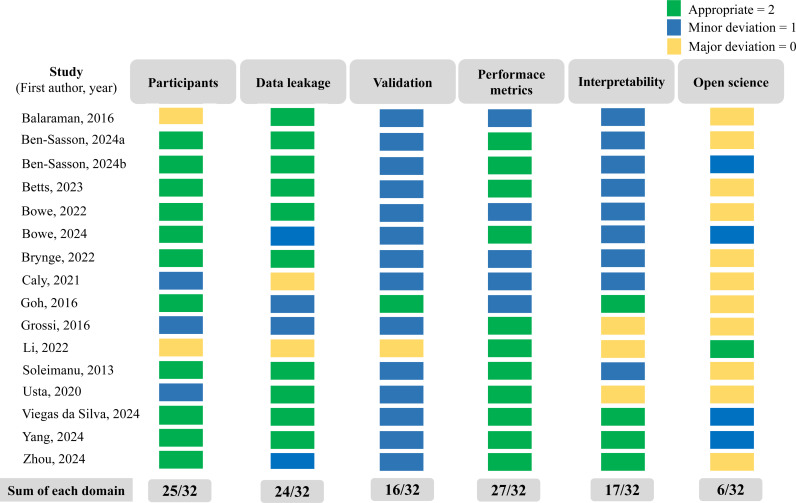
Summary of the quality assessment of ten prediction-focused ML studies. This figure provides an overview of the quality assessment of 16 prediction-focused ML studies focused on child development. The first author and publication year are listed on the left. Six domains of the evaluation criteria are displayed from left to right: participants, data leakage, validation, performance metrics, interpretability, and open science. The sum scores for each study are detailed in Table 2. The aggregated scores for each domain across all studies are shown in light gray boxes at the bottom of the figure. Color coding indicates the level of quality: green represents appropriate, blue indicates minor deviations, and yellow highlights major deviations.

In Table 2, we report the quality of each prediction-focused ML study according to the criteria described in the Methods Section. Across the 16 prediction-focuses ML studies, the total study quality ranges from a low score of 5 to a high score of 10 (full score = 12, mean score = 7.2, standard deviation = 1.8), suggesting typical study qualities are moderate. In Fig 3, we present the total scores for each of the six different quality-criteria across 16 studies. If all ten studies had completely met the criteria, the corresponding total score for the criteria would have resulted in a full score of 32. Across the six criteria, total scores range from 6 (open science) to 27 (performance metrics). The remaining four criteria show moderate quality. All observed studies suffered from some limitations in either the procedures to properly validate results or avoid data leakage. The specific issues were different in each case, ranging from missing or incomplete splitting strategies to issues of data leakage. At the same time, all studies reported at least some common performance metrics. Most studies, however, indicated metrics only for a fixed classification threshold value, implying a specific choice of the trade-off between precision and recall that was not discussed or motivated explicitly. Four studies addressed this issue by providing AUC values, underscoring the value of considering the full range of trade-offs represented in the ROC curve. These findings highlight a need for future prediction-focused ML studies to adopt more robust validation practices.

Most studies in the criterion of open science show major deviations, with only one study providing most of the related code and data. In fact, the majority of studies provide no disclosure on the availability of data and code. The apparent reservation to making more technical details available might be a further indication of the experimental state of the methodology. Overall, the results indicate that the existing studies may not yet provide a clear template or best-practice example to inform future research. Interestingly, for each criterion, there is at least one example that appropriately fulfills the criteria. As these positive examples occur across very different basic approaches, it would not be obvious though, how they could be combined into a single study design in the future.

## Discussion

This review offers an overview of how ML is applied in studies that employ statistical models to analyze maternal exposure to risk factors and child development. The majority of the included studies have a descriptive focus on the associations between perinatal factors and child development outcomes. Additionally, 26.7% of the prediction-focused ML studies employed predictive ML models for supporting differential diagnoses. Among the ML techniques reported, Decision Tree Models and Artificial Neural Networks were the most prevalent.

While the pattern-focused ML studies discussed in this review have provided valuable insights into potential relationships within complex biological, psychosocial, and environmental systems, their limitations in establishing causality must be acknowledged, particularly in their capacity to influence medical routines and interventions. The inherent nature of association studies to identify correlations does not equate to causation. This restricts their direct applicability in developing preventive and therapeutic strategies [123]. In the pursuit of precise identification of early markers or predictors in developmental pathways, as stated in our introduction, the reliance solely on association studies can risk overlooking critical causal factors. Association studies are prone to various biases, such as selection bias and confounding, which can lead to spurious conclusions [124]. Therefore, a shift towards more prediction-oriented research, and ideally, studies that establish causality, is paramount. Causal studies, supported by predictive analytics, would provide a more robust framework for discerning the complex interplay of factors at work.

The evaluation of prediction-focused ML studies reveals several quality indicators, including sample sizes, interpretability, data leakage, and open science, showing minor to major deviations. Across ten studies, several quality indicators, including sample sizes, interpretability, data leakage, and open science, show minor to major deviations. In other words, most of the results need to be interpreted with caution due to several methodological limitations. First, studies based on large-scale populations and longitudinal datasets in healthcare are still limited [125]. Studies with sample sizes of less than 100 mother-child dyads and a large number of predictors might suffer from overfitting, which might not be detected in the absence of external validation metrics [55,75]. Second, potential limitations of the representativeness of the samples should be also noted. Third, in prediction models, standardized internal validation and model evaluation are lacking. Fourth, perinatal factors and child outcomes are subject to changes over time. Most studies focused on a particular time period and the results might not be replicable due to the dynamic nature of data. Fifth, there is a general lack of transparency. Possibly due to privacy concerns or sensitive data, most studies do not provide information and materials according to open science guidelines [126]. Overall, ML models are suitable in identifying correlations and statistical inferences based on complex and large datasets. For prediction modelling observed in the current review, however, the methodology remains immature, with limitations in sample size, validation, transparency, and adaptability. On a positive note, over the past two years, there has been a notable increase in publications on ML, encompassing both pattern-based and prediction-based approaches. Among prediction-based ML studies published after 2023, a positive trend was that all reported appropriate performance metrics and offered meaningful model interpretations. However, we did not observe any substantial improvements in other quality criteria and the ML techniques used in prediction-based studies continued to vary widely.

The challenges for ML applications we describe are consistent with several recent reviews. For instance, in the fields predicting population health and developmental outcomes, the methods of hyperparameter selection, number of feature selection, and methods of feature selection, are often under-reported [127,128]. Moreover, similar to our observations, small samples are frequently used [17,128]. Another common issue is the difficulty to assess data quality, data leakage, and the handling of missing data. In the healthcare sector, these challenges are particularly pronounced due to the sensitive nature of patient information and the complexity of medical data. ML techniques are increasingly being used to address these issues. Ensuring data quality involves using advanced algorithms to validate and clean data, maintaining accuracy, completeness, and consistency, which are crucial for reliable clinical outcomes [129]. Data leakage in ML occurs when information that should not be available to the training dataset inadvertently influences model creation, leading to overly optimistic performance estimates that fail to generalize to new data [130]. This issue often arises when practitioners use ML tools without fully understanding the underlying algorithms and nature of their datasets, relying instead on automated processes [131]. Understanding and mitigating data leakage is crucial for developing robust and reliable ML models that can generalize effectively to real-world scenarios [130,131]. Additionally, handling missing data effectively is vital to avoid biases in research findings. ML methods like Multiple Imputation by Chained Equations (MICE), K-Nearest Neighbors (KNN), and Denoising Autoencoders (MIDAS) have shown promise in accurately imputing missing data, ensuring that healthcare datasets remain robust and useful for improving patient care and advancing medical research [129]. Furthermore, nontransparent reporting of how data are handled might contribute to low validity and prediction accuracy of the reported prediction models [132]. Future studies therefore should report more details of the selection and data processing procedure. With clear and transparent information, though sample sizes for training are small, the results are more likely to further increase the predictive performance of larger future datasets.

Besides increasing transparency, several areas of improvement could be explored in future research. For the pattern-focused ML studies using the BKMR approach, the analysis could be extended for the purpose of prediction. For this purpose, a validation scheme based on a corresponding split of the dataset would be necessary. However, potential hurdles for adopting BKMRs for research projects beyond the specific domain of modeling of multi-pollutant mixtures might be their limitation to continuous variables, the sensitivity of posterior inclusion probabilities to the choice of tuning parameters, and the lack of procedures for assessing the statistical significance of pollutants mixture interactions [133,134].

For prediction-focused ML studies, beyond well-defined special cases, future research will still face a challenging landscape of competing ML techniques with distinct advantages and disadvantages. In ML research, it is well-established that RFMs and other decision-tree based models demonstrate high performance, even when the amount of available data is limited [135]. However, recent advances in transfer learning could make the use of more expressive ANNs feasible even for small datasets [136]. For this approach, a neural network is trained on a large, often domain-general dataset and then fine-tuned on a specific smaller dataset. Whether transfer learning can be effectively applied to prediction-focused ML studies such as those discussed in this review remains an open question that could be addressed by future research.

Finally, across both prediction- and pattern-focused ML studies, a range of maternal external and internal risk factors for child development have been identified. This review specifically examines maternal risk factors throughout pregnancy and the first 12 months postpartum. Capturing the dynamics and changes over time in the mother is inherently complex due to the multifaceted nature of maternal health, behavior, and environment. Similarly, child development is a highly dynamic and intricate process, influenced by a myriad of factors that evolve over time. Future research challenges will involve not only capturing these parallel complexities but also understanding the interplay between maternal and child development. This will require sophisticated longitudinal studies and advanced analytical methods, such as ML techniques, to unravel the intricate web of influences that shape early childhood development.

## Conclusions

This scoping review shows that a majority of the observed studies have employed ML techniques for statistical associations between perinatal factors and their impact on child outcomes. Among prediction-focused ML studies, (Decision Tree-based) Ensemble Algorithms and Artificial Neural Networks were the most frequently utilized ML techniques. In contrast to inferential findings, the interpretability and generalizability of prediction studies are more limited. While several performance metrics were reported, to fully meet the evaluation criteria used in the quality assessment, it will be necessary to develop a more systematic methodology and enhance the transparency of future research.

## Limitations

There are some limitations in the current scoping review. First, the literature search was limited to PubMed, Scopus, and Web of Science, with Google Scholar used as a supportive tool. Studies from unpublished data, thesis work, or grey literature were not included, potentially limiting the comprehensiveness of the findings. Second, while some included studies had relatively small sample sizes, the review does not aim to determine the optimal sample size for studies in this field. However, the variability in sample sizes across studies could influence the robustness and generalizability of the findings. Third, the quality assessment of the studies was limited to prediction-focused ML studies. Finally, the studies included were highly heterogenous, with considerable variation in ML techniques, timing of exposure, outcome measures, and child development assessment tools. This heterogeneity makes it difficult to draw conclusive results regarding the overall impact of perinatal factors on child developmental problems.

## Supporting information

S1 Fig Distribution of different outcomes across 16 prediction-focused ML studies. (PDF)

S2 Fig Distribution of different outcomes across 44 pattern-focused ML studies. (PDF)

S1 Table Boolean, search strategies. (DOCX)

S2 Table PRISMA checklist. (DOCX)

S3 Table Quality assessment. (DOCX)
