# Supplementary figures and images for: A scoping review and quality assessment of machine learning techniques in identifying maternal risk factors during the peripartum phase for adverse child development
Source: PLoS One. 2025 May 28;20(5):e0321268. doi: 10.1371/journal.pone.0321268 (PMC12119027; doi:10.1371/journal.pone.0321268)

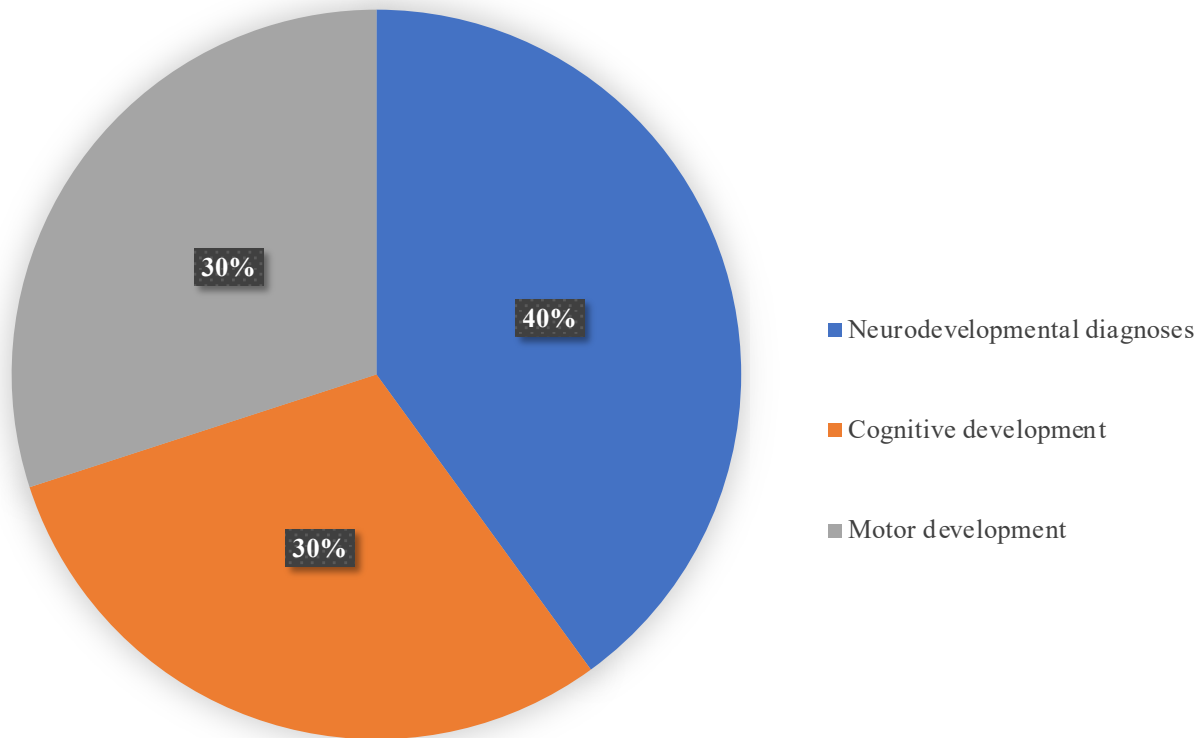

**S1 Fig.** Distribution of different outcomes across 16 prediction-focused ML studies.

Supplement: S1 Fig — (PDF) [file pone.0321268.s001.pdf]

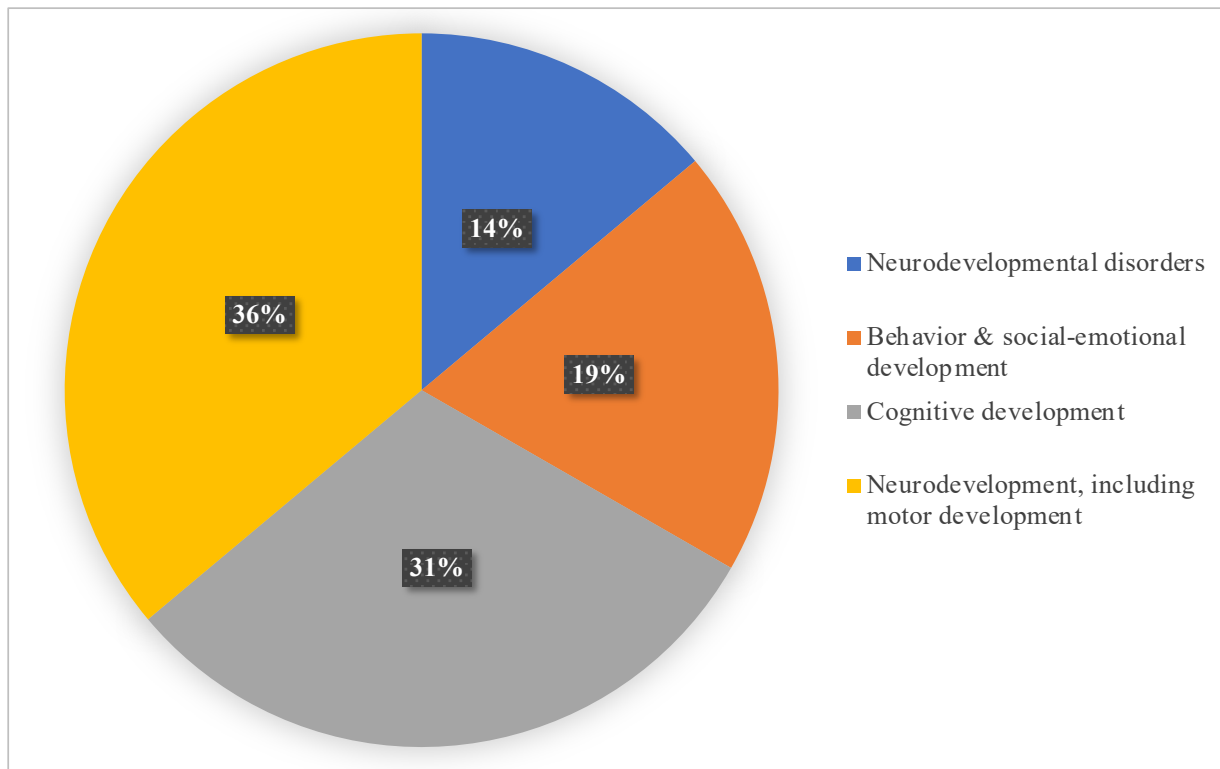

**S2 Fig.** Distribution of different outcomes across 44 pattern-focused ML studies.

Supplement: S2 Fig — (PDF) [file pone.0321268.s002.pdf]
