# Supplementary material for: A scoping review and quality assessment of machine learning techniques in identifying maternal risk factors during the peripartum phase for adverse child development
Source: PLoS One. 2025 May 28;20(5):e0321268. doi: 10.1371/journal.pone.0321268 (PMC12119027; doi:10.1371/journal.pone.0321268)
Supplement: S1 Table — (DOCX) [file pone.0321268.s003.docx]

Supplementary Table 1. Boolean used in literature search

| **Keyword Group I** | | **Keyword Group II** | **Keyword Group III** |
| --- | --- | --- | --- |
| perinatal, antenatal, prenatal, postnatal factors, postpartum, and pregnancy | | infant outcome, newborn, motor development, cognitive development, regulation, social emotional development | machine learning, artificial intelligence, artificial neural networks, BKMR, decision tree, deep learning, K-mean, neural network, random forest, regression tree, supervised learning, semi-supervised learning, and unsupervised learning |
|  | | | |
| **Search Boolean** | | | |
|  | | | |
| Google Scholar | | | |
| “intitle:infant cognitive development AND perinatal factor AND machine learning -review -protocol -preterm”, “intitle:infant social emotional development AND perinatal factor AND machine learning -review -protocol -preterm”, and “intitle:infant motor development AND perinatal factor AND machine learning -review -protocol -preterm” | | | |
|  | | | |
| Line # | PubMed NLM | | |
| 1 | "Pregnancy"[Mesh:NoExp] OR "Postpartum Period"[Mesh:NoExp] | | |
| 2 | perinatal[Title/Abstract] OR antenatal[Title/Abstract] OR prenatal[Title/Abstract] OR postnatal[Title/Abstract] OR postpartum[Title/Abstract] OR pregnanc*[Title/Abstract] | | |
| 3 | #1 OR #2 | | |
| 4 | "Infant"[Mesh:NoExp] OR "Infant, Newborn"[Mesh:NoExp] OR "Child Development"[Mesh:NoExp] OR "Cognition"[Mesh] OR "Prenatal Exposure Delayed Effects"[Mesh] | | |
| 5 | infant*[Title/Abstract] OR newborn*[Title/Abstract] OR child development[Title/Abstract] OR cognition[Title/Abstract] OR motor development[Title/Abstract] OR cognitive development[Title/Abstract] OR regulation[Title/Abstract] OR social emotional development[Title/Abstract] OR (“prenatal exposure”[tiab] AND delayed effect*[tiab]) | | |
| 6 | #4 OR #5 | | |
| 7 | "Bayes Theorem"[Mesh] OR "Neural Networks, Computer"[Mesh] OR "Algorithms"[Mesh:NoExp] OR "Artificial Intelligence"[Mesh:NoExp] OR "Machine Learning"[Mesh] OR "Decision Trees"[Mesh] OR natural language processing[mesh] OR sentiment analysis[mesh] | | |
| 8 | Bayes theorem[Title/Abstract] OR algorithm*[Title/Abstract] OR decision tree*[Title/Abstract] OR machine learning[Title/Abstract] OR Artificial intelligence[Title/Abstract] OR deep learning[Title/Abstract] OR random forest[Title/Abstract] OR BKMR[Title/Abstract] OR Bayesian Kernel Machine Regression[Title/Abstract] OR K-mean*[Title/Abstract] OR neural network*[Title/Abstract] OR regression tree*[Title/Abstract] OR semisupervised learning[Title/Abstract] OR semi supervised learning[Title/Abstract] OR natural language processing[tiab] OR sentiment analysis[tiab] | | |
| 9 | #7 OR #8 | | |
| 10  studies | (#3 AND #6 AND #9 AND english[la] AND 2023/03/01:2025/12/31[dp]) NOT (Systematic Review[publication type] OR Meta-Analysis[publication type] OR Cochrane Database Syst Rev[ta] OR ((“systematic review”[tiab:~3]) OR meta analys*[tiab] OR metaanalys*[tiab])) | | |
|  | | | |
| Line # | Web of Science Core Collection | | |
| 1 | TS=(Perinatal OR antenatal OR prenatal OR postnatal OR postpartum OR pregnanc*) | | |
| 2 | TS=(infant* OR newborn* OR “child development” OR cognition OR “motor development” OR “cognitive development” OR regulation OR “social emotional development” OR (“prenatal exposure” AND “delayed effect*”)) | | |
| 3 | TS=(“Bayes theorem”OR algorithm* OR “decision tree*” OR “machine learning” OR “Artificial intelligence” OR “deep learning” OR “random forest” OR BKMR OR “Bayesian Kernel Machine Regression” OR “K-mean*” OR “neural network*” OR “regression tree*” OR “semisupervised learning” OR “semi supervised learning” OR “natural language processing” OR “sentiment analysis”) | | |
| 4 | #1 AND #2 AND #3 | | |
| Studies | ***Filter: Article*** *(Document Types) and* ***English*** *(Languages) Timespan: 2023-03-01 to 2025-12-31 (Index Date)* | | |
|  | | | |
| Line # | Scopus | | |
| 1 | TITLE-ABS-KEY(Perinatal OR antenatal OR prenatal OR postnatal OR postpartum OR pregnanc*) | | |
| 2 | TITLE-ABS-KEY(infant* OR newborn* OR “child development” OR cognition OR “motor development” OR “cognitive development” OR regulation OR “social emotional development” OR (“prenatal exposure” AND “delayed effect*”)) | | |
| 3 | TITLE-ABS-KEY(“Bayes theorem”OR algorithm* OR “decision tree*” OR “machine learning” OR “Artificial intelligence” OR “deep learning” OR “random forest” OR BKMR OR “Bayesian Kernel Machine Regression” OR “K-mean*” OR “neural network*” OR “regression tree*” OR “semisupervised learning” OR “semi supervised learning” OR “natural language processing” OR “sentiment analysis”) | | |
|  | #1 AND #2 AND #3 | | |
| 4 | #1 AND #2 AND #3 AND PUBYEAR > 2023 AND PUBYEAR < 2026 AND ( LIMIT-TO ( DOCTYPE , "ar" ) ) AND ( LIMIT-TO ( SRCTYPE , "j" ) ) AND ( LIMIT-TO ( LANGUAGE , "English" ) ) | | |
