# Supplementary material for: A scoping review and quality assessment of machine learning techniques in identifying maternal risk factors during the peripartum phase for adverse child development
Source: PLoS One. 2025 May 28;20(5):e0321268. doi: 10.1371/journal.pone.0321268 (PMC12119027; doi:10.1371/journal.pone.0321268)
Supplement: S3 Table — (DOCX) [file pone.0321268.s005.docx]

Supplementary Table 3. Quality assessment criteria and results

| **First author** | **Participants** | **Data leakage** | **Validation** | **Performance metrics** | **Interpretability** | **Open science** |
| --- | --- | --- | --- | --- | --- | --- |
| Balaraman (2016) | MA | A^1^ | MI | MI | MI | MA |
| Ben-Sasson (2024) | A | A^1^ | MI | A | MI | MA |
| Ben-Sasson (2024) | A | A^1^ | MI | A | MI | MI |
| Betts (2023) | A | A | MI | A | MI | MA |
| Bowe (2022) | A | A^1^ | MI | MI | MI | MA |
| Bowe (2024) | A | MI | MI | A | MI | MI |
| Brynge (2022) | A | A^1^ | MI | MI | MI | MA |
| Caly (2021) | MI | MA | MI | MI^5^ | MI | MA |
| Goh (2016) | A | MI^2^ | A | MI^6^ | A | MA |
| Grossi (2016) | MI | MI^1,3^ | MI | A | MA | MA |
| Li (2022) | MA | MA^1,4^ | MA | A | MA | A |
| Soleimani (2013) | A | A^1^ | MI | A | MI | MA |
| Usta (2020) | MI | A^1^ | MI | A | MA^7^ | MA |
| Viegas da Silva (2024) | A | A^1^ | MI | A | A | MI |
| Yang (2024) | A | A^1^ | MI | A | A | MI |
| Zhou (2024) | A | MI | MI | A | A | MA |
| **Criteria of the rating** | A: sample size > 300  MI: sample size 100 to 300  MA: sample size < 100 | Based on predictor selection and data leakage A: there was no data leakage and predictor selection and model selection were independent from validated or cross-validated results  MI: there was clear data leakage and predictor selection but model selection was independent from validated or cross-validated results, or vice versa  MA: there was clear data leakage and predictor selection and model selection was not independent from validated or cross-validated results | A: both internal and external validation (or cross-validation, or splitting up data into dedicated training and testing sets) were performed  MI: only internal or external validation (or cross-validation, or splitting up data into dedicated training and testing sets) was performed  MA: absence of internal validation and external validation, cross-validation, or splitting up data into dedicated training and testing sets | A: Area under the receiver operating characteristics curve (AUC) or combination of other metrics for different prediction thresholds was reported  MI: only some metrics were reported, e.g., sensitivity analysis  MA: none of the above-mentioned was reported | A: both interpretability of the model and comparison with previous models were performed to a great extent; or interpretability of the model was performed to a great extent and comparison with previous models were performed to some extent  MI: interpretation of the model was performed to some extent  MA: No interpretable model reported and no explicit interpretation provided | A: free online availability of code, model, and data and/or provision of a decision support tool  MI: limited sharing of code, model, or data (or only available upon request)  MA: No sharing of code, model, or data and no decision support tool |
| Abbreviations: A, appropriate; MI, minor deviation; MA, major deviation.  ^1^The authors did not provide detailed information related to hyperparameter tuning.  ^2^MI was rated due to the unexplained results regarding almost equal performance between training and validation.  ^3^MI was rated due to the unclear information regarding Twist approach.  ^4^This study did not use a train and test splitting approach and the result of the prediction was based on the existing data.  ^5^There was no report of “Area under the **receiver operating characteristics** curve (AUC)” and was not threshold agnostics.  ^6^AUC was not reported but others several metrics were reported.  ^7^Unclear information on decision tree model types. | | | | | | |
